# Neutrophil-only Histological Assessment of Ulcerative Colitis Correlates with Endoscopic Activity and Predicts Long-term Outcomes in a Multicentre Study

**DOI:** 10.1093/ecco-jcc/jjad110

**Published:** 2023-06-30

**Authors:** Tommaso L Parigi, Rosanna Cannatelli, Olga M Nardone, Irene Zammarchi, Uday Shivaji, Federica Furfaro, Davide Zardo, Paola Spaggiari, Rachele Del Sordo, Orsola Setti, Snehali Majumder, Samuel C L Smith, Silvio Danese, Alessandro Armuzzi, Vincenzo Villanacci, Subrata Ghosh, Marietta Iacucci

**Affiliations:** Institute of Immunology and Immunotherapy, University of Birmingham, Birmingham, UK; Department of Immunology, Transplantation and Infectious Diseases, University Vita-Salute San Raffaele, Milan, Italy; Institute of Immunology and Immunotherapy, University of Birmingham, Birmingham, UK; Gastroenterology Unit, Department of Biomedical and Clinical Sciences ‘L. Sacco’, University of Milan, Milan, Italy; Institute of Immunology and Immunotherapy, University of Birmingham, Birmingham, UK; Gastroenterology, Department of Public Health, University of Naples Federico II, Naples, Italy; Dipartimento Medicina Interna e Terapia Medica, University of Pavia, Pavia, Italy; Gastroenterology Unit, ASST-Spedali Civili Brescia, Brescia, Italy; Institute of Immunology and Immunotherapy, University of Birmingham, Birmingham, UK; University Hospitals Birmingham NHS Foundation Trust, Gastroenterology, Birmingham, UK; Department of Gastroenterology and Digestive Endoscopy, IRCCS Ospedale San Raffaele, Milan, Italy; Department of Pathology, San Bortolo Hospital, Vicenza, Italy; Pathology Unit, IRCCS Humanitas Research Hospital, Rozzano, Milan, Italy; Department of Medicine and Surgery, Section of Anatomic Pathology and Histology, Medical School, University of Perugia, Perugia, Italy; Institute of Pathology, ASST-Spedali Civili Brescia, Brescia, Italy; Institute of Immunology and Immunotherapy, University of Birmingham, Birmingham, UK; Institute of Immunology and Immunotherapy, University of Birmingham, Birmingham, UK; University Hospitals Birmingham NHS Foundation Trust, Gastroenterology, Birmingham, UK; Department of Immunology, Transplantation and Infectious Diseases, University Vita-Salute San Raffaele, Milan, Italy; Department of Gastroenterology and Digestive Endoscopy, IRCCS Ospedale San Raffaele, Milan, Italy; Department of Biomedical Sciences, Humanitas University, Pieve Emanuele, Milan, Italy; IBD Center, IRCCS Humanitas Research Hospital, Rozzano, Milan, Italy; Institute of Pathology, ASST-Spedali Civili Brescia, Brescia, Italy; College of Medicine and Health, University College Cork, and APC Microbiome Ireland, Cork, Ireland; Institute of Immunology and Immunotherapy, University of Birmingham, Birmingham, UK; College of Medicine and Health, University College Cork, and APC Microbiome Ireland, Cork, Ireland; Division of Gastroenterology, NIHR Birmingham Biomedical Research Centre, Birmingham, UK

**Keywords:** Inflammatory bowel diseases, neutrophils, PHRI, PICaSSO, histological remission, endoscopic remission

## Abstract

**Backgrounds and Aims:**

Absence of neutrophils is the minimum standard to consider histological remission of ulcerative colitis [UC]. The PICaSSO Histological Remission Index [PHRI] is a new simple index for UC, based only on the detection of neutrophils. We evaluate PHRI’s correlation with endoscopy and its prognostic value compared with other established indices.

**Methods:**

Consecutive patients with UC underwent colonoscopy at two referral centres [Birmingham, UK, and Milan, Italy,] and were followed up for 2 years. Correlation between histology (PHRI, Nancy [NHI], and Robarts [RHI] indexes) and endoscopy (Mayo Endoscopic Score [MES], Ulcerative Colitis Endoscopic Index of Severity [UCEIS], and PICaSSO index) was calculated as Spearman coefficients. Diagnostic performance of endoscopy was assessed with receiver operating characteristic [ROC] curves and outcome stratification with Kaplan–Meier curves.

**Results:**

A total of 192 patients with UC was enrolled, representing all grades of endoscopic severity. Correlation between histology and endoscopy did not differ significantly when using PHRI instead of NHI or RHI. In particular, PHRI’s correlation with MES, UCEIS, and PICaSSO was 0.745, 0.718, and 0.694, respectively. Endoscopically-assessed remission reflected the absence of neutrophils [PHRI = 0] with areas under the ROC curve of 0.905, 0.906, and 0.877 for MES, UCEIS, and PICaSSO, respectively. The hazard ratio for disease flare between patients in histological activity/remission was statistically similar [p >0.05] across indexes [2.752, 2.706, and 2.871 for RHI, NHI, and PHRI, respectively].

**Conclusion:**

PHRI correlates with endoscopy and stratifies risk of relapse similarly to RHI and NHI. Neutrophil-only assessment of UC is a simple yet viable alternative to established histological scores.

## Graphical Abstract

**Graphical Abstract:**
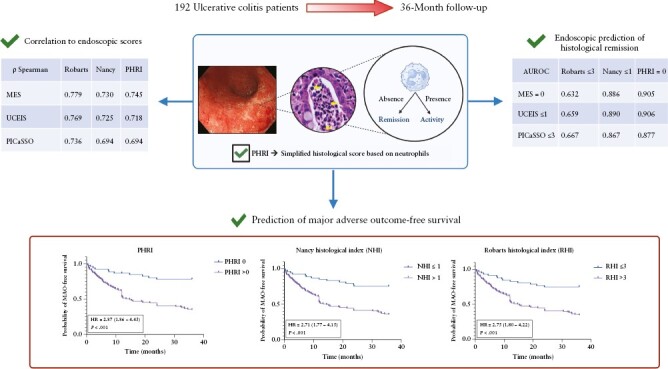

## 1. Introduction

Current management of ulcerative colitis [UC] relies on an objective assessment of disease activity. Endoscopy is the mainstay of UC evaluation,^1,2^ but growing evidence shows that inflammation persistence at histological level, even in absence of macroscopic signs of disease, carries important prognostic implications.^3^ Accordingly, recommendations have started considering histological remission as a desirable target of treatment^4^ and clinical trials, starting from the ustekinumab phase 3 UNIFI study,^5^ are including histological remission as a secondary outcome measure. Our group has previously shown how part of the discrepancy between endoscopy and histology can be overcome using virtual chromoendoscopy [VCE] and a dedicated scoring system, the Paddington International virtual ChromoendoScopy ScOre [PICaSSO].^6,7^ However, the widespread scoring of endoscopic severity in daily practice relies on simpler indexes such as the Mayo Endoscopic Score [MES] that, albeit imperfect, is easily remembered and requires no specific training.^8^

Numerous histological indexes have been developed to standardise assessment of UC severity, but unlike in endoscopy, their use in daily practice is minimal and limited to research settings.^9,10^ Hence the prognostic value of histology remains largely undervalued. This shortcoming is partly due to the complexity of most scoring systems, which are time-consuming for pathologists and require adequate training and experience. Because most indexes include subjective measures, the interobserver variability is high even among expert pathologists^11^ and higher among non-experts.^12^

We previously demonstrated how neutrophilic infiltration, the hallmark of UC activity devoid of additional histological features, strongly correlates with endoscopy and disease course.^13^ As part of the multicentre PICaSSO study, six expert pathologists developed a score based only on the presence or absence of neutrophils, the PICaSSO Histological Remission Index [PHRI], intended to reduce subjectivity and simplify assessment. PHRI’s details are provided in Supplementary Table 1. The index was then tested on more than 600 biopsies and compared with five other histological indices (Robarts’[RHI], Nancy [NHI], Geboes, ECAP, and Villanacci scores) for correlation with endoscopy [assessed as MES, UCEIS, PICaSSO, and the vascular and mucosal sub-scores of PICaSSO], prediction of disease flare, and interobserver variability. In that study, PHRI showed a statistically stronger correlation with endoscopy and prognostic value, and high interobserver agreement.^13^ For these reasons, we proposed PHRI as a practical option for the histological assessment of UC, overcoming at once complexity and subjectivity and providing useful prognostic information. Importantly, due its simplicity, it has been possible to successfully implement PHRI into artificial intelligence models that replicate human assessment of UC histology.^13,14^

Around the same time when PHRI was developed, scientific societies, in an effort to provide guidance on histological evaluation of UC activity, recommended the use of NHI and RHI, the two most rigorously validated scores, and in parallel agreed that absence of neutrophils should be considered as the minimum standard for histological remission.^10,15^ Importantly, PHRI’s definition of remission [absence of neutrophils and of erosions and ulcers] perfectly matches the recommended criteria by the European Crohn’s and Colitis Organisation [ECCO],^10^ and could therefore serve as a practical minimum standard, at least in daily practice. However, PHRI is still very new and lacks external validation. In the present study, we aim to externally validate it on a large cohort of UC patients, assessing its correlation with endoscopy and its ability to predict the occurrence of flare during follow-up.

## 2. Methods

We conducted a post hoc analysis of data from three prospective studies conducted in two tertiary referral centres in Birmingham, UK, and Milan, Italy. The studies’ protocols were approved by the research ethics committee of each centre [CARMS n.14392 and Ref 17/NI/0148 for Birmingham] and [ethics approval n.2678 for Milan]. These cohorts were previously reported in other publications.^16,17^

### 2.1. Patients

Adult patients [age 18 to 75] with an established diagnosis of UC for at least 1 year, undergoing colonoscopy for disease assessment or surveillance, were prospectively enrolled between April 2018 and November 2020. Exclusion criteria were inability to provide consent, unclassified colitis, Boston Bowel Preparation Scale Score <2 in the examined colonic segment, and contraindications to endoscopy or biopsies. All procedures were recorded and performed by experienced endoscopists in Birmingham and Milan. Demographic, clinical, endoscopic, and histological data were collected at the time of endoscopy [baseline] and at up to 36 months of clinical follow-up.

### 2.2. Endoscopic assessment

Endoscopy was performed using high-definition [HD] Narrow Band Imaging [NBI] [290 series, Olympus, Tokyo, Japan] or HD, linked colour imaging [LCI], and blue light imaging [BLI] [Lasereo system, FujiFilm, Tokyo, Japan]. The activity of the disease was assessed in white light with the Mayo Endoscopic Score [MES]^8^ and Ulcerative Colitis Endoscopic Index of Severity [UCEIS]^18^ and with virtual chromoendoscopy through the PICaSSO score.^19^ Endoscopic remission was defined as MES = 0, UCEIS ≤1, and PICaSSO ≤ 3.

### 2.3. Histological assessment

**Figure 1.**
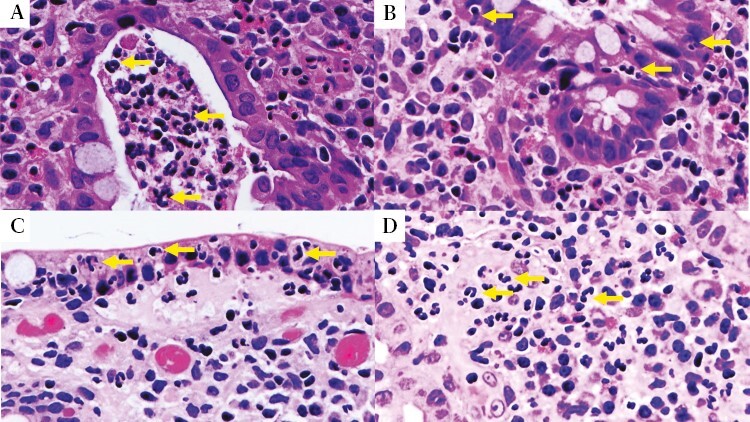
Examples of neutrophilic infiltration. A] Crypt abscess [arrows on neutrophils] H&E × 40. B] Neutrophils infiltrating the cryptal epithelium [arrows on neutrophils] H&E × 40. C] Neutrophils in the superficial epithelium [arrows on neutrophils] H&E × 40. D] Neutrophils in lamina propria [arrows on neutrophils] H&E × 40. H&E, haematoxylin and eosin.

At least two targeted biopsies were taken from the worst inflamed areas. Samples were fixed in formalin, processed at the two centres, and stained with haematoxilin and eosin. Histological activity was assessed using RHI^20^ and NHI^21^ by two pathologists expert in IBD pathology [DZ in UK; PS and VV in Italy]. The Italian pathologist [VV], experienced with PHRI, reviewed all the samples and scored the PHRI for all biopsies. Histological remission was defined as NHI ≤1,^21^ or as RHI ≤3 without neutrophils in the epithelium or lamina propria,^22^ or as PHRI = 0 [absence of neutrophils from superficial epithelium and lamina propria].^13^ Pathologists were blinded to clinical and endoscopic data [Figure 1].

### 2.4. Clinical outcome

The following pre-specified major adverse outcomes [MAO] were manually retrieved from electronic medical records at 12, 24, and 36 months: colectomy due to drug-refractory UC [excluding colectomies due to dysplasia or cancer], UC-related hospitalisation, need for steroids, change or addition of UC treatment due to uncontrolled inflammation, and dose or frequency increase of an ongoing biologic/advanced small molecule due to ongoing inflammation [excluding development of immunogenicity]. In case more MAOs occurred around the same time [ie, hospital admission, steroid course, and colectomy] the first in order of importance was considered, as listed above [colectomy > hospitalisation > steroids > treatment change > treatment optimisation]. For survival analysis, the first major adverse outcome was considered.

### 2.5. Statistical analysis

Data were stored in Microsoft Excel and analysed using GraphPad [GraphPad Software, San Diego, US]. Continuous variables were expressed as mean ± standard deviation [SD] and analysed by Mann–Whitney test or Kruskal–Wallis test. Percentages were calculated and Fisher’s exact test or chi square statistics were used.

Sample size analysis was based on the primary endpoint of histological-endoscopic correlation. The effect size was estimated based on our previous work^13^ that found correlation coefficients between the two measures around 0.7. Hence, power calculation with arctan transformation, one-sided, with r = 0.5 and alpha = 0.05, resulted in a optimal sample size of 23, well within our sample availability.

The strength of the correlation between histological and endoscopic scores was measured as Spearman’s correlation coefficient. Coefficients of 0.8–1.0 were considered as ‘very strong’, 0.6–-0.79 as ‘strong’, 0.4–-0.59 as ‘moderate’, and 0.2–-0.39 as ‘weak’. Spearman coefficients were compared with Games–Howell pairwise comparison test.

To assess endoscopic scores’ diagnostic performance for histological remission, ROC [receiver operating characteristic] curves were plotted and the areas under the ROC curve [AUROC] were calculated. AUROCs were compared using DeLong test. Outcome analysis was performed and Kaplan–Meier for probability of major adverse outcome [MAO]-free survival were plotted. Patients with no follow-up data were excluded from the outcome analysis and patient with incomplete follow-up were censored after the date of their last visit.

## 3. Results

### 3.1. Patient characteristics

A total of 192 UC patients were included [111 enrolled in Birmingham, UK, and 81 in Milan, Italy]. The mean age at baseline was 44.1, 98 [51%] were female, average disease duration was 11.6 years [SD 9.5], 59% had extensive colitis, 34% left-sided colitis, and 6% proctitis. All grades of endoscopic severity were represented. Patients’ characteristics at baseline are summarised in Table 1, and major clinical outcomes are detailed in Table 2. Most clinical outcomes were related to medication start or addition [29], switch or swap from one biologic or advanced small molecule to another [18], or dose/frequency optimisation [22]. No dose increase was reported among patients on advanced small molecules.

**Table 1.** Patient demographics and characteristics at baseline.

Characteristics	Patients [192]
Age [y] mean [SD]	44.09 [14.7]
Gender female *n* [%]	98 [51%]
Extension of disease *n* [%]	
Proctitis	12 [6.2%]
Left colitis	66 [34.4%]
Pancolitis^**^	114 [59.4%]
Disease duration [y] mean [SD]	11.6 [9.5]
Endoscopic activity	
Mayo 0	66 [34.4]
Mayo 1	44 [22.9]
Mayo 2	48 [25.0]
Mayo 3	34 [17.7]
Therapy at baseline	
No treatment	10 [5.2%]
5-ASA	86 [44.8%]
Corticosteroids	23 [12.0%]
Immunosuppressants	31 [16.1%]
Biologics/advanced small molecules	42 [21.9%]
Robarts Histological Index mean [SD]	9.7 [9.9]
Nancy Histological Index mean [SD]	1.9 [1.4]
PHRI mean [SD]	1.8 [1.4]

SD, standard deviation; y, years; 5-ASA, 5-aminosalicylate; PHRI, PICaSSO Histological Remission Index.

^**^Ulcerative colitis extending proximal to the splenic flexure.

**Table 2.** First major adverse clinical outcome [MAO] recorded per patient.

Major adverse outcome [MAO]	Patients
Total	91
Colectomy	11
Hospitalisation^a^	2
Steroids need^b^	9
UC medication^c^ start or addition	29
Biologic or advanced small molecule swap	18
Dose/frequency escalation^d^	22

UC, ulcerative colitis.

^a^Excluding those for, or resulting in, colectomy.

^b^Excluding steroids prescribed during hospitalisation.

^c^Excluding steroids.

^d^Not due to immunogenicity.

### 3.2. Primary endpoint

The correlation between endoscopic and histological scores measured through Spearman coefficients was strong. In particular, PHRI correlation with MES, UCEIS, and PICaSSO was 0.74, 0.72, and 0.69, respectively; NHI‘s correlation with the same endoscopic measures was 0.73, 0.72, and 0.69, respectively; and RHI’s was 0.78, 0.77, and 0.74, respectively. All comparisons of correlation coefficients between histological indexes were statistically non-significant [p >0.05], meaning that the choice of the histological index did not impact on correlation with endoscopy Table 3.

**Table 3.** Spearman correlation coefficients.

Endoscopic score	Robarts	Nancy	PHRI
MES	0.779	0.730	0.745
UCEIS	0.769	0.725	0.718
PICaSSO	0.736	0.694	0.694

PICaSSO, Paddington International virtual ChromoendoScopy ScOre; PHRI, PICaSSO Histological Remission Index; MES, Mayo Endoscopic Score; UCEIS, Ulcerative Colitis Endoscopic Index of Severity.

### 3.3. Secondary endpoints

Endoscopic assessment through different scores and respective cut-offs predicted the underlying histological remission defined as PHRI = 0. The area under the ROC curve for MES, UCEIS, and PICaSSO was 0.905 (95% confidence interval [CI] 0.864–0.947), 0.906 [95% CI 0.866–0.947], and 0.877 [95% CI 0.828–0.926], respectively. DeLong testing for AUROC comparison for endoscopic scores show–ed no statistical difference [p >0.05].

The same analysis using RHI’s and NHI’s definitions of histological remission resulted in numerically smaller AUROCs for all endoscopic scores, MES, UCEIS, and PICaSSO [Table 4; and Supplementary Figure 1].

**Table 4.** Area under the ROC for endoscopy’s prediction of histological remission.

AUROC [95% CI]	PHRI = 0	NHI ≤ 1	RHI ≤ 3*
**MES = 0**	0.905 [0.864–0.947]	0.886 [0.837 - 0.934]	0.632 [0.553 - 0.711]
**UCEIS ≤ 1**	0.906 [0.866–0.947]	0.890 [0.842 - 0.938]	0.659 [0.582 - 0.736]
**PICaSSO ≤ 3**	0.877 [0.828–0.926]	0.867 [0.814 - 0.921]	0.667 [0.591 - 0.744]

AUROC, area under receiver operating curve; PICaSSO, Paddington International Virtual ChromoendoScopy ScOre; PHRI, PICaSSO Histological Remission Index; MES, Mayo Endoscopic Score; UCEIS, Ulcerative Colitis Endoscopic Index of Severity; NHI, Nancy Histological Index; RHI, Robarts Histological Index; CI, confidence interval.

### 3.4. Stratification of clinical outcomes with histology

**Figure 2.**
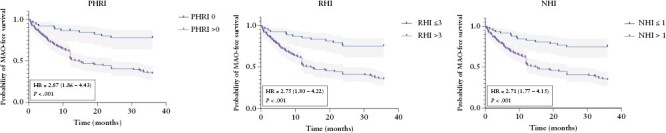
Kaplan–Meier curves for risk of major adverse outcomes [MAO] during follow-up.

Of the total 192 patients, 14 [7%] were excluded from outcome analysis due to lack of any follow-up data. Baseline characteristics of these patients are reported in Supplementary Table 2; of note, 10 out of 14 had MES 0 at endoscopy. Histological assessment provided good stratification of risk of MAO [colectomy, hospitalisation, change of treatment, or treatment optimization], proxies for disease flare. At survival analysis, the hazard ratio between patients histologically active and in remission according to PHRI, RHI, and NHI were 2.87 [95% CI 1.86–4.43], 2.75 [95% CI 1.79–4.22], and 2.71 [95% CI 1.77–4.15], respectively, with no statistical difference [*p* >0.05] between the three [Table 5 and Figure 2].

**Table 5.** Hazard ratios for disease flare between patients in remission or activity according to endoscopy, histology, and combined endoscopic plus histological remission vs non-remission.

Hazard ratios	Endoscopy only	RHI ≤3*	NHI ≤1	PHRI = 0
**Histology-only**		2.75 [1.79–4.22]	2.71 [1.77–4.15]	2.87 [1.86–4.43]
MES = 0	3.00 [1.97–4.59]	3.13 [2.00–4.88]	3.13 [2.00–4.88]	3.05 [1.95–4.78]
UCEIS ≤1	2.96 [1.94–4.53]	2.91 [1.85–4.59]	2.84 [1.81–4.46]	2.91 [1.89–4.59]
PICaSSO ≤3	2.73 [1.80–4.14]	3.04 [1.96–4.71]	3.01 [1.94–4.67]	3.20 [2.05–4.99]

PICaSSO, Paddington International Virtual ChromoendoScopy ScOre; PHRI, PICaSSO Histological Remission Index; MES, Mayo Endoscopic Score; UCEIS, Ulcerative Colitis Endoscopic Index of Severity; NHI, Nancy Histological Index; RHI, Robarts Histological Index; CI, confidence interval.

### 3.5. Stratification of clinical outcomes with endoscopy

Patients with endoscopic activity, according to MES, had a hazard ratio of 3.00 [95% CI 1.97–4.59] of suffering a major adverse outcome; if classified according to UCEIS, the hazard ratio was 2.96 [95% CI 1.94–4.53], and according to PICaSSO was 2.73 [95% CI 1.80–4.14]. All comparisons of risk of flare stratification between histological and endoscopic assessments were non-significant [p > 0.05], though hazard ratios were numerically higher for endoscopy score [Table 5].

### 3.6. Prediction of clinical outcomes with combined histological and endoscopic remission

When combining endoscopic and histological remission, the stratification did not improve significantly as compared with the prognostic value of each assessment considered individually. The hazard ratios for all the scores combinations are reported in Table 5.

## 4. Discussion

The uptake of standardised histological grading of UC activity remains minimal in daily practice.^9^ The difficulty of available indexes and their interobserver variability are two of the main reasons why histological remission is still not commonly used as a target of treatment, despite its association with better long-term outcomes^23,24^ and cancer prevention.^25^

Recent international guidelines on UC histopathology proposed absence of intraepithelial neutrophils, erosions, and ulceration as the minimum standard for the definition of histological remission.^10,15^ The PHRI score was developed around the same time as these guidelines through a consensus process based on the empirical observation that neutrophils held the strongest correlation with endoscopy and clinical outcome. In other words, the pathologists who developed PHRI reached the same conclusion as the guidelines: neutrophil infiltration explains the greatest part of the variance between assessments and the clinical implication of histological inflammation. In the rare event that erosions and ulcers are visible in absence of neutrophils, according to PHRI they should be scored as 1, meaning equivalent to activity. However, this circumstance never occurred in the biopsies in the present study. Therefore, we propose that PHRI can fulfil the task of minimum standard for histological assessment as recommended by scientific societies, and its ease of use may permit wider adoption. The benefits of this approach are several. A single variable [presence or absence of neutrophils] reduces the subjectivity of interpretation, requires no additional training for the pathologist, and does not imply extra work other than standard assessment of the biopsy. In the present work we provide strong evidence that histological remission, simply defined as lack of neutrophil infiltration [PHR =  0], is similar to remission defined on the basis of NHI and RHI. Moreover, we confirmed association of PHRI with endoscopy, the latter assessed both in white light and in VCE [PICaSSO]. Correlation coefficients of PHRI with MES, UCEIS, and PICaSSO were between moderate and good, broadly similar to those found in the original PHRI development study.^13^ Of note, contrary to our previous observation, assessment with VCE and the PICaSSO score did not result in a significantly stronger correlation with histology. Our results further confirm that with the use of HD scopes and VCE, endoscopic assessment is getting closer to histology.^26^

When looking at the ability of endoscopy to predict histological remission, we observed a similar diagnostic performance for the three endoscopic scores. Contrary to previous studies,^7^ including some from our group, these results suggest that endoscopic scoring did not affect the recognition of underlying histological activity. Importantly, endoscopy with either score performed numerically better for the diagnosis of histological remission defined by PHRI [AUROCs range 0.906 to 0.877] followed by NHI [AUROCs range 0.867 to 0.890] and RHI [AUROCs range 0.632 to 0.667], further suggesting that inflammatory changes seen in endoscopy, regardless of the score, are mainly associated with neutrophilic infiltration.

The prognostic value of PHRI, meaning its ability to stratify patients’ risk of flare during follow-up, was similar to that of RHI and NHI. This observation supports the initial hypothesis that absence of neutrophils, regardless of other histological features, is a sufficiently accurate measure of disease remission and therefore a clinically useful criterion. Instead, the combination of endoscopic and histological remission did not improve stratification of outcomes [Table 5] as compared with the two assessments separately. This is likely due to the study population. In fact, we included patients with all severities of disease and, although unintended, there was a roughly similar distribution of severity grades, with 42.7% of patients with Mayo 2 or 3 endoscopic activity. Because the additional benefit of histological remission in outcome prediction is more evident in patients with endoscopic remission or mild activity [ie, Mayo 0–1], in our heterogeneous population the expected gain from histology was likely ‘diluted’. Overall, combined endo-histological endpoints are still exploratory, their operational performance in different contexts needs to be further investigated, and as our group previously demonstrated, advanced endoscopy and dedicated scoring can reduce the gap between endoscopy and histology.^7,26^

To our knowledge, this is the first study to externally validate the first neutrophil-only score PHRI. Broadly, our work demonstrates the potential use of neutrophil-only assessment in clinical practice to simplify scoring while maintaining a good correlation with endoscopic activity and outcome stratification. In other words, absence of neutrophils is a good and easy proxy for deep remission, as shown by the low rates of relapse.

The study has some limitations. Follow-up data were not available for all patients and this could theoretically introduce selection bias if more unwell patients had been lost to follow-up. Nevertheless, we believe this risk is negligible because both hospitals are large tertiary referral centres with experience in complex cases, and there is no indication that the loss to follow-up was greater among patients in histological activity or remission. Our prognostic analysis focused on assessment of endoscopic and histological activity or remission in a dichotomous fashion, therefore not considering the whole spectrum of severity. However, histology is mainly relevant to UC prognosis in the case of mild or quiescent disease, whereas it adds less information to a clearly inflamed endoscopic picture. Moreover, the correlation analysis did include the full breadth of all scores and again found no difference among them. Unlike RHI and NHI which were scored by the pathologists of each centre at the time of cohort enrolments, PHRI—which was not yet developed at the time—was later scored by a single pathologist [VV] who did not participate in the original studies. For this reason we could not assess interobserver variability among pathologists; however, we have investigated it in our previous work.^13^

Besides, our group has previously demonstrated how PHRI can be successfully implemented in an artificial intelligence system to expedite and automate histological assessment.^13,25^ Programming a computer to detect a single type of cell is far easier than having it replicate a complex human assessment based on numerous features. Digital pathology is already available in several centres, and at the current pace of digitalisation, it will become ubiquitous in the near future. Hence, delegating a computer to provide the activity assessment could become as easy as pressing a button.

We believe that neutrophil-only assessment of UC disease activity is the necessary compromise to overcome histological complexity and increase uptake of standardised scoring in clinical practice. Limiting the assessment to neutrophils, using PHRI, provides a practical solution. Moreover, PHRI allows also to grade inflammation and thus to evaluate treatment response, which may be complicated by features such as architectural changes or eosinophilia, less reflective of current inflammation and more influenced by the previous course of disease.

In conclusion, neutrophil-only assessment of UC histological activity with PHRI is a reliable option to validate scores which provides a similar correlation with endoscopy and prognostic stratification.

## Supplementary Material

jjad110_suppl_Supplementary_Figure_S1

jjad110_suppl_Supplementary_Table_S1

jjad110_suppl_Supplementary_Table_S2

## Data Availability

Data underlying this article will be shared on reasonable request to the corresponding author.
