# Supplementary material for: Neutrophil-only Histological Assessment of Ulcerative Colitis Correlates with Endoscopic Activity and Predicts Long-term Outcomes in a Multicentre Study
Source: J Crohns Colitis. 2023 Jun 30;17(12):1931–8. doi: 10.1093/ecco-jcc/jjad110 (PMC10798862; doi:10.1093/ecco-jcc/jjad110)
Supplement: jjad110_suppl_Supplementary_Table_S1 [file jjad110_suppl_supplementary_table_s1.docx]

**Supplementary Table 1.**

**The PICaSSO Histologic Remission Index (PHRI)**

| **Histologic Finding** | **Score** |
| --- | --- |
| **Neutrophil infiltration in lamina propria** |  |
| Absent (No) | 0 |
| Present (Yes) | 1 |
| **Neutrophil infiltration in epithelium** |  |
| Absent (No) | 0 |
| Present (Yes) |  |
| - Surface epithelium^#^ | 1 |
| - Cryptal epithelium (cryptitis) | 1 |
| - Crypt abscess | 1 |
| **Total Score** = sum of all above (maximum 4)* |  |

**Criteria** for the scoring histologic components:

1. “*Neutrophil infiltration”* (in either lamina propria or epithelium): Any number (even only one) of neutrophil(s) is acceptable. (Evaluation under high power view at 40x is required if “absence of neutrophil” is determined. Neutrophil in the lamina propria must be outside of capillary lumina.)
2. *“Crypt abscess”*: cryptitis with any number of neutrophils or any amount of neutrophilic exudate overflowing into cryptal lumen AND any degree of cryptal epithelial cell injury.

^#^ If a biopsy has no intact surface epithelium but shows features of erosion/ulceration ( *e.g*., granulation tissue, or/and inflammatory exudates), also score 1.

*When there are multiple biopsies from different segments of bowel, the maximum/highest/worst score (PHRI_max) among all biopsy sites will be the preferred ‘*global score’*.

_____________________________________________

Adapted from Gui X, Bazarova A, Del Amor R, et al. Gut 2022 May;71(5):889-898.
