# Supplementary material for: Neutrophil-only Histological Assessment of Ulcerative Colitis Correlates with Endoscopic Activity and Predicts Long-term Outcomes in a Multicentre Study
Source: J Crohns Colitis. 2023 Jun 30;17(12):1931–8. doi: 10.1093/ecco-jcc/jjad110 (PMC10798862; doi:10.1093/ecco-jcc/jjad110)
Supplement: jjad110_suppl_Supplementary_Table_S2 [file jjad110_suppl_supplementary_table_s2.docx]

| **Characteristics** | **Patients (14)** |
| --- | --- |
| Age (y) *mean (SD)* | 48 (14.3) |
| Gender Female n (%) | 6 (43 %) |
| **Extension of disease n (%)** |  |
| Proctitis | 1 (7%) |
| Left colitis | 9 (64%) |
| Pancolitis^**^ | 4 (29%) |
| Disease duration (y) mean (SD) | 11.3 (7.6) |
| **Endoscopic activity** |  |
| Mayo 0 | 10 (71.4) |
| Mayo 1 | 1 (7.1) |
| Mayo 2 | 2 (14.3) |
| Mayo 3 | 1 (7.1) |
| **Histological activity at baseline** |  |
| Robarts Histological Index mean (SD) | 3.14 (3.1) |
| Nancy Histological Index mean (SD) | 1.2 (1.2) |
| PHRI mean (SD) | 0.64 (0.84) |

**Supplementary Table 2.**

Baseline characteristics of patients excluded from the outcome analysis due to missing follow-up
