# Supplementary material for: Neutrophil-only Histological Assessment of Ulcerative Colitis Correlates with Endoscopic Activity and Predicts Long-term Outcomes in a Multicentre Study
Source: J Crohns Colitis. 2023 Jun 30;17(12):1931–8. doi: 10.1093/ecco-jcc/jjad110 (PMC10798862; doi:10.1093/ecco-jcc/jjad110)

PHRI - ROC curve

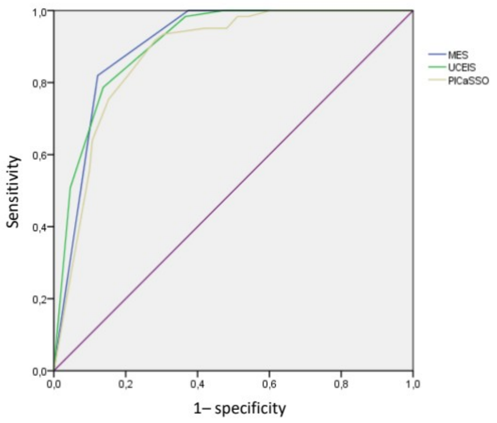

RHI - ROC curve

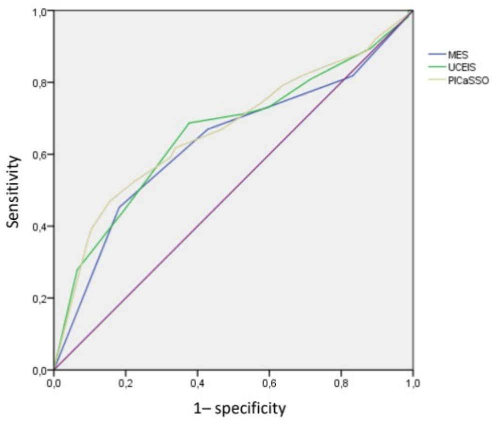

RHI - ROC curve

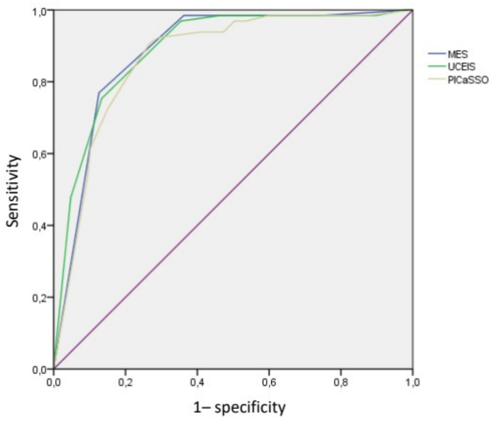

Supplement: Supplementary file 3 [file jjad110_suppl_supplementary_figure_s1.docx]
